# Transactions of the American Institute of Homœopathy

**Published:** 1888-01

**Authors:** 


TRANSACTIONS OF THE AMERICAN INSTITUTE OF HOMŒOPATHY, in its Fortieth Session. Edited by Dr. J. C. Burgher, General Secretary, 1887.

To publish this portly volume of eight hundred and eighty-six pages so promptly is indeed a credit to the Secretary. As to the matter contained in this volume, it is as usual a sad mixture, with little in it that would please the eye of a Hahnemann or a Hering! Still there are some good things; the discussions and papers upon defective sleep and infantile eczema are interesting, although the treatment which seemed most popular with the members would hardly commend itself to more earnest homœopaths.

